# New Appliances and Things Medical

**Published:** 1900-02-24

**Authors:** 


### NEW APPLIANCES AND THINGS MEDICAL.

**[We shall be glad to receive, at our Office, 28 & 29, Southampton Street, Strand, London, W.C., from the manufacturers, specimens of all new preparations and appliances which may be brought out from time to time.]**

### IODINE VASOGEN.

(PEARSON AND CO., HAMBURG. SOLE AGENT FOR THE UNITED KINGDOM, E. J. REID, 11 DUNEDIN HOUSE, BASINGHALL STREET, LONDON, E.C.)

VASOGEN is a variety of oxygeneated vaseline, a form of mineral oil which is peculiarly adapted for inunction, since not only is it very readily absorbed by the skin, but also because, owing to its solvent properties, it constitutes a first-class vehicle for many bodies which are not soluble in water. Iodoform, menthol, eucalyptol, iodine, and various alkaloids and antiseptics have been combined with vasogen and introduced into the system by means of inunction. Iodine has, however, been more extensively used in this manner than any other drug—within a few hours after inunction it can be demonstrated in the urine. It is, in fact, so rapidly absorbed that little or no discolouration of the skin ensues, and for this reason is well adapted for application to visible parts of the integument. For glands in the neck and goitre it answers capitally. It is also of great value for all conditions in which iodides or iodine are indicated—in syphilis, certain forms of bronchitis, and arterio-sclerosis. Iodine vasogen is prepared in three strengths, *i.e.*, 6 per cent., 10 per cent., and 20 per cent. The first-named is probably the most suited for the majority of cases.

### PATENT TOE SPRING.

(POND, CASTLE MEADOW, NORWICH.)

THIS new patent toe spring is intended for the relief and cure of deformed toes and bunions. It is a small steel apparatus covered with washleather, and fitted to the great and first toe, and held in position by elastic, which passes over and under the foot and round the heel. When fixed in position the great toe is kept in line with the inside of the foot, or as nearly in line as anatomical relations will allow; the first toe is also straightened out to a certain extent. The apparatus is only to be worn at night, and is not altogether very uncomfortable. We are not, however, prepared to admit that such an apparatus, or any apparatus, can effect the cure of a bunion, nor correct osseous deformity of the great toe joint when once it has been established, although in young persons such an apparatus as that patented by Pond, if worn consistently during a considerable length of time, may assist to correct any early tendency to the development of a bunion.

### PLASMON.

(THE PLASMON SYNDICATE, 56, DUKE STREET, GROSVENOR SQUARE, LONDON, W.)

PLASMON is a most interesting food product, not merely from the fact that it is practically a new departure, but also from the importance, from a culinary point of view, of having at hand a practically tasteless proteid, by aid of which foods of many kinds can be "strengthened" irrespective of the character of their own flavour. Plasmon is, we are told, derived from skimmed or separated milk, which in many country districts is very cheap. The introduction of machinery, by which milk can be entirely deprived of its cream, which is by far the most valuable part of it, has led to a large quantity of skim milk being left almost as a bye-product upon the dairyman's hands. Often this goes to the pigs, which, indeed, is a very handy way of turning it into a more saleable form of food. The inventor of plasmon has, however, invented a better way. He precipitates the albuminous or proteid portion of the milk, but instead of allowing the precipitated casein to ferment as in cheese-making, he at once adds to the separated curd sufficient bicarbonate of soda to replace the alkali which has been lost in the process of coagulation. By this means, and by the subsequent kneading process which it undergoes by machinery in an atmosphere of carbonic acid, it is reduced to a soluble powder. The result of this ingenious process is that instead of, as in old days, the whole contents of the milk-pail being turned into cheese of varied flavour and of varied degrees of digestibility, the modern centrifugator removes the cream, which is quickly turned into a valuable and easily-transportable food in the form of butter, while the above-mentioned process quickly turns the albuminous or proteid portion of the milk into a portable and permanent food product in the form of plasmon. Practically, then, except the water and some of the sugar, nothing is thrown away. This so-called "plasmon" consists of the proteid of milk, free from fat and sugar, but containing a certain proportion of mineral salts, largely the phosphates precipitated from the milk along with the curd. In character plasmon is a light, friable powder, without odour and without flavour, which is soluble in water, in milk, and other aqueous liquids, such as soup. In warm water it may be dissolved in quantities up to 30 per cent., which is about the percentage in which albumin exists in meat. Such a soluble proteid food as plasmon, prepared as it is directly from milk, ought to be of considerable dietetic value, taking the place of other albuminous compounds; and from the accounts given of the various careful dietetic experiments which have been made by chemists and physiologists such seems to be the case. In fact, from the simplicity of the process by which it is derived from the freshly-separated milk we should entertain no doubt about its digestibility and ready assimilability, even if we had not had these reports before us, and as to its keeping power and general convenience there can be no question. There seems every reason to believe that plasmon will take a very important place among "foods," especially as it consists entirely of the thing in which farinaceous and vegetable-derived foods are mostly lacking, namely, proteid.

